# Gambling treatment service providers’ views about contingency management: a thematic analysis

**DOI:** 10.1186/s12954-022-00600-0

**Published:** 2022-02-25

**Authors:** Lucy Dorey, Darren R. Christensen, Richard May, Alice E. Hoon, Simon Dymond

**Affiliations:** 1 School of Psychology, Swansea University, Singleton Campus, Swansea, SA2 8PP UK; 2 Faculty of Health Sciences, University of Lethbridge, Lethbridge, AB Canada; 3 University of Melbourne, Parkville, VIC 3010 Australia; 4 School of Psychology and Therapeutic Studies, University of South Wales, Pontypridd, CF371DL UK; 5 Swansea University Medical School, Singleton Campus, Swansea, SA2 8PP UK; 6 Department of Psychology, Reykjavík University, Menntavegur 1, Nauthólsvík, 101 Reykjavík, Iceland

**Keywords:** Contingency management, Gambling, Treatment, Thematic analysis, Qualitative

## Abstract

**Background:**

There is a need to improve retention and outcomes for treatment of problem gambling and gambling disorder. Contingency management (CM) is a behavioural intervention involving identification of target behaviours (such as attendance, abstinence, or steps towards recovery) and the provision of incentives (such as vouchers or credits towards the purchase of preferred items) contingent on objective evidence of these behaviours. Contingency management for abstinence and attendance in substance misuse treatment has a substantial evidence base but has not been widely adopted or extended to other addictive behaviours such as gambling. Potential barriers to the widespread adoption of CM may relate to practitioners’ perceptions about this form of incentive-based treatment. The present study sought to explore United Kingdom (UK) gambling treatment providers’ views of CM for treatment of problem gambling and gambling disorder.

**Methods:**

We conducted semi-structured interviews with 30 treatment providers from across the UK working with people with gambling problems. Participants were provided with an explanation of CM, several hypothetical scenarios, and a structured questionnaire to facilitate discussion. Thematic analysis was used to interpret findings.

**Results:**

Participants felt there could be a conflict between CM and their treatment philosophies, that CM was similar in some ways to gambling, and that the CM approach could be manipulated and reduce trust between client and therapist. Some participants were more supportive of implementing CM for specific treatment goals than others, such as for incentivising attendance over abstinence due to perceived difficulties in objectively verifying abstinence. Participants favoured providing credits accruing to services relevant to personal recovery rather than voucher-based incentives.

**Conclusions:**

UK gambling treatment providers are somewhat receptive to CM approaches for treatment of problem gambling and gambling disorder. Potential barriers and obstacles are readily addressable, and more research is needed on the efficacy and effectiveness of CM for gambling.

**Supplementary Information:**

The online version contains supplementary material available at 10.1186/s12954-022-00600-0.

## Background

Gambling disorder (GD) is an addictive disorder characterised by recurrent, problematic gambling and leads to significant harm across several life domains including financial difficulties, relationship breakdown, emotional distress, and health deterioration [1]. Rates of lifetime GD and subthreshold ‘problem gambling’ range internationally between 0.12% and 5.8% [2]. In 2016, for instance, it was estimated that 0.7% of the population of England, 0.8% of Wales and 1% of Scotland experienced gambling-related problems in the past year (i.e. they scored 8 or more on the Problem Gambling Severity Index, PGSI) [3].

Treatment options for reducing the harms caused by GD, while readily accessible in some jurisdictions (e.g. the National Gambling Treatment Service in Great Britain) and with a growing evidence base [4], are rather disparate. Blank et al. [4] recently identified eleven systematic reviews (including three meta-analyses) between 2012 and 2019 of psychotherapeutic interventions for problem gambling. The reviews compared psychotherapeutic interventions either with each other, to a peer aid/mutual recovery condition (e.g. Gamblers Anonymous), or to a waiting list control condition, on a range of outcomes such as gambling problem severity and frequency of gambling. The authors found evidence for the short-term effectiveness of behavioural interventions (e.g. exposure therapy), Cognitive Behavioural Therapy (CBT), which involves identifying triggers and coping strategies, and uses techniques such as cognitive reappraisal, CBT combined with Motivational Interviewing (MI), which combines a client-centred empathic style with directive interventions to promote behaviour change, and Brief Interventions for less severe problems (usually 1–3 sessions including aspects of MI). Evidence for the long-term effectiveness of these interventions is limited, however, and more research is needed to understand the impact of treatment on the different domains of gambling-related harm.

Opportunities for identification of people with gambling problems through screening within GP and mental health services are often missed [5, 6]. It is estimated that only 10% of those with GD seek psychological treatment [5] but the number that receive treatment may in fact be much lower. For instance, 9,008 individuals were recorded as having received treatment from the National Gambling Treatment Service in Great Britain in 2019 [6], which suggests that less than 5% of adults with GD actually encountered gambling treatment services [7]. Moreover, between 20 and 50% discontinue treatment prematurely or dropout once they have started [8]. Recently, Pfund et al. [9] reviewed the overall prevalence of dropout from treatment for GD and problem gambling and found a weighted dropout rate of 39.1% (95% CI [33.0%, 45.6%]). Dropout rates were significantly higher among non-married clients and when dropout was defined as anything other than attending all sessions of a prescribed treatment protocol. In Great Britain, estimates suggest that 26.2% of those who start treatment dropout before completion [10], while many other individuals are likely to leave between initial assessment and the commencement of treatment, yet such figures are not currently reported. Increasing the rates of individuals who begin treatment and then remain in treatment until completion are important challenges for face-to-face psychological treatment services for GD and problem gambling. Continued attendance at treatment sessions is significantly associated with improved therapeutic outcomes [9], and a great deal more research is needed on how to enhance treatment completion.

Contingency management (CM) is an empirically validated behavioural therapy for promoting behaviour change in areas such as alcohol and substance-use disorders, medication adherence, and other health-related behaviours [11–13]. The application of CM procedures has been shown to improve treatment retention in substance-use treatment programmes and vaccination-session attendance and uptake among individuals with complex needs [14–16]. Contingency management is based on principles of operant conditioning and involves the identification of target behaviours (such as attendance at therapy, abstinence from gambling, or completing steps towards one’s recovery goals), and the provision of tangible reinforcers (often monetary-based such as vouchers or credits towards the purchase of preferred items) when evidence of the target behaviour is produced [17]. According to principles of operant reinforcement, occurrence of the target behaviour should increase when it is followed by the reinforcer. In this way, CM fosters new habits and the replacement from other sources of reinforcement for the target behaviour. CM may hold promise for increasing retention in treatment for GD and problem gambling by incentivising session attendance, gambling abstinence and, ultimately, recovery.

There is substantial evidence from other domains showing that CM targeting attendance (ATT-CM) increases treatment retention [14]. Pfund et al. [16] conducted a meta-analysis of ATT-CM alongside substance misuse treatment (10 studies; *n* = 1841) and found a moderate effect on attendance (*d* = 0.47, 95% CI [0.25, 0.69]), and a small effect on abstinence (*d* = 0.22, 95% CI [0.12, 0.33]). To illustrate how ATT-CM was implemented in one of those studies, Petry et al. [14] recruited adults with cocaine use disorder starting treatment (*n* = 360) and randomised participants to usual care (UC), or 6 or 12 weeks of UC plus ATT-CM. Attendance at each group treatment session was incentivised by inclusion in a prize draw: on attending each scheduled therapy session, participants could win small, medium, or large prizes (worth up to $100.00 in value). Findings showed that attendance was significantly higher in the 12 weeks ATT-CM condition compared to UC (*d* = 0.54, 95% CI [0.39, 0.88]), but not for 6 weeks of ATT-CM. There is comparable meta-analytic evidence of the effectiveness of CM in targeting abstinence from illicit drug use (ABS-CM) [11]. For example, Dutra et al. [18] found a moderate-to-large effect size (*d* = 0.58, 95% CI [0.25, 0.90]), while a recent meta-analysis of studies, with up to a year follow up after ending CM, found increased likelihood of abstinence in the CM group (OR 1.22, 95% CI [1.01, 1.44]) [19]. Taken together, the meta-analytic evidence suggests that CM has a moderate-to-large effect on increasing attendance and decreasing dropout in substance misuse settings [11, 18, 20]. Despite this, the implementation of CM and its extension to gambling treatment have been slow.

To our knowledge, there is currently only one pilot randomised control trial evaluating the impact of CM as a treatment component for GD [15]. In this ongoing study, which is being conducted by one of the present authors, 54 treatment-seeking gamblers meeting DSM-V criteria for GD were recruited from sites across rural Canada and randomly assigned to either a CBT plus CM group or a CBT-alone group. Participants in the CM + CBT group can gain credits for attendance at online therapy sessions, presenting financial evidence of gambling abstinence (e.g. bank statements), family member corroboration of abstinence, and study completion. The number of credits earned increases when goals are achieved on consecutive weeks, reduce back to starting levels when goals are not attained, but can be redeemed to the prior achieved level again after 3 consecutive weeks of abstinence. The maximum credits earned in 12 weeks are $C450 and can be exchanged for goods or services online or converted to a gift card for local services. Data collection is ongoing, having been impacted by COVID-19, and thus, we must await the full findings, but preliminary analyses indicate a beneficial impact of CM on both treatment attendance and gambling abstinence.

To facilitate dissemination of CM as a potential new adjunct treatment for GD, it is important to consider the perspectives of treatment providers and clients as to their perceptions of CM. In related studies of potential barriers to the implementation of CM for substance use disorders, Kirby et al. [21] developed the *Provider Survey of Incentives* (PSI), based on in-depth interviews with treatment service providers in the USA. The aim of the PSI was to identify and quantify the frequency of specific beliefs about CM, which Kirby et al. administered to a sample of 383 treatment providers. These authors found that a high percentage of participants (45–74%) agreed with the 9 positive statements endorsing the use of CM. Practitioners most often agreed with concerns that CM would be unaffordable (43–74% depending on monthly cost between $10 and $150), would not address the underlying issues of addiction (52%), and that rewarding abstinence alone (50%) or attendance alone (42%) would not be right. Concerns about jealousy between clients (39%) and of drawing people into treatment for the wrong reasons (33%) were also commonly endorsed. Other items less frequently endorsed (20–30%) related to concerns about CM being labour intensive, having a negative impact on motivation, not providing long lasting effects, clashing with personal philosophy, or being seen as cheesy by clients. Further studies in other jurisdictions like the US criminal justice system [22] and substance use treatment settings in Australia [23] and the UK [24] have identified similar overlapping concerns. Some differences were noted such as greater support for individualised approaches to CM according to need (‘vertical equity’) in UK substance use treatment contexts [24], while Australian practitioners were more open to providing CM in a harm reduction context, without requiring evidence of abstinence [23], and practitioners with prior exposure to CM had more favourable views [25].

It is possible that practitioners’ concerns can be addressed through education and training in the rationale and methodology of CM, providing supervision, and adapting CM to the broader treatment context [11]. Indeed, Rash et al. [26] found that workshops provided for practitioners working with veterans significantly changed attendees' perceptions of CM, improving participants’ post intervention preparedness to implement CM. There are potential benefits of applying CM to gambling. If CM can improve retention in treatment, then clients are likely to benefit from participating in a greater number of therapy sessions, leading to better outcomes [27]. Similarly, if CM can increase periods of abstinence and establish new recovery behaviours, then this could improve long-term treatment outcomes and aid full recovery. Despite this, implementation of CM is still slow and, apart from Christensen et al.’s work [15], arguably absent from current treatment approaches for GD and problem gambling [11]. Moreover, no data currently exist as to the primary concerns that UK-based therapists and clients may hold about this treatment for GD.

The broad aim of the present study was to investigate the feasibility of adding CM to existing psychological treatments for GD in the UK. As a first step, our specific aim is to answer the question: “What do providers of gambling treatment services perceive as potential benefits of, and barriers to, the implementation of CM for gambling?” To address this, we developed and conducted a survey and in-depth follow up interviews of UK gambling treatment service providers’ views of CM as an adjunct treatment for GD generally, and for increasing treatment retention and abstinence from gambling, specifically.

## Methods

### Participants and design

We employed a pragmatic and qualitative approach using cross-sectional surveys and semi-structured interviews as part of a larger study. We recruited staff aged over 18 years old who were responsible for delivering treatment or providing advice and support for people with gambling problems in England, Scotland, and Wales. A purposive sampling approach was used [28]; we aimed to recruit 30 participants with at least five people from each of the following types of service-providers: managers/senior clinicians, frontline advice workers, and therapists/counsellors. These groups are collectively referred to as ‘practitioners’ in this article. All participants received a £15 Amazon voucher on completion of the study. Ethical approval was received from the School of Psychology Research Ethics Committee at Swansea University (20th November 2020).

### Data collection

After distributing a letter to several gambling treatment and frontline advice service providers in England, Scotland, and Wales, six sites indicated willingness to support recruitment. A contact person at each site circulated recruitment emails to eligible staff and further promotion of the study took place via social media. Potential participants were provided with a Participant Information Sheet and further information on the study website [29] and were invited to book a research appointment via an online booking system.

The first author (LD) conducted the interviews and analysis drawing on her professional background as an addictions practitioner and qualitative methods training undertaken alongside her PhD studies. Participants were not known to LD prior to the initiation of the interviews, except where site managers were contacted prior to the study to assist in recruitment. Participants were briefly informed of LD’s background. Semi-structured interviews lasting one and a half hours were conducted and audio recorded online in Zoom or Teams software between 8 December 2020 and 22^nd^ February 2021; only the researcher and participant were present and there were no follow-up interviews.

The topic guide (see Additional file 1) and interview process were first piloted with an individual with lived experience of GD and with one practitioner. Participants were given the opportunity to ask any questions about taking part in the study and then completed an online consent form and demographic and background information hosted by Qualtrics (Table 2). Participants were first provided with a brief description of CM, and then asked:

- In your opinion, do you feel this could be a successful approach in treatment for people who want help for their gambling problem?
- Would you be interested in delivering such a programme if it became available?
- What type of barriers to success do you think this type of approach might encounter?

Two scenarios (adapted from 21, 24) were then presented and participants were asked to discuss the strengths and weaknesses of both approaches (included in Topic Guide, see Supplementary Materials). In scenario 1, clients receive credits (exchangeable for goods or services with staff assistance) for sharing bank statements absent of any evidence of gambling activity, with credits increasing with consecutive occasions of providing proof of abstinence. In scenario 2, clients receive vouchers (exchangeable for food or goods) for attendance, with increasing rewards for consecutive attendance.

Participants then completed the PSI and *Contingency Management Adoption Attitudes* (CMAA) scale [30], with minor adjustments to the gambling treatment setting (see Supplementary Materials) and were invited to comment further on questions they had responded to as ‘strongly agree/disagree’, or any other item. This semi-structured approach was helpful to facilitate in-depth discussion of a topic with which most participants had no prior knowledge. Field notes were made by LD following the interviews.

### Approach to analysis

Interview audio data were transcribed and checked by LD using NVivo Transcription online service. Transcripts were not checked or further commented on by participants. Data were analysed using Reflexive Thematic Analysis (Reflexive TA), an approach suited for understanding participant views of a topic [31]. The six stages of Reflexive TA are familiarisation, generating codes, constructing themes, revising themes, defining themes, and producing the report. Throughout analysis, LD drew on her theoretical understanding of the range of interventions applied to addictions, and knowledge of the literature related to CM. A pragmatic philosophical position, oriented towards solving problems in the world [32] was adopted within the reflexive TA framework. Familiarisation with the data highlighted differences between the views of participants about CM for gambling and the literature on practitioner views of CM for substance misuse. Thus, complete inductive coding rather than selective deductive coding was used to capture the full range of perspectives in relation to gambling. The coding tree is provided in Table 1. During analysis, a fifteen-point checklist for good Thematic Analysis was followed [28, p. 287]. Coding and initial analysis were carried out by one researcher (LD) and after ten transcripts had been coded, AH reviewed several transcripts to add another perspective to the development of the coding structure [33]. No novel codes were identified from later interviews, suggesting the sample size was adequate. Themes were developed from across the codes, bringing together interrelated aspects of the data [34]. Themes were discussed by the authors and within a project steering group including people with lived experience of gambling problems.

**Table 1:** Coding tree with examples of child codes

Group of codes	Root codes	Child codes examples
***Participant approach and overall view of CM***	Models applied to gambling	
	Overall view of CM	
	Past experience of CM or similar	
***Cost and cost effectiveness***	Cost is a potential barrier	
	CM affordable	
	Gambling industry money	
	I don’t know how effective CM is	
	Ideas for funding sources	
	It’s not my role to say	
	Labour intensive/time consuming	
	Probity	
***Moral or ethical issues***	Clients should be doing this anyway	
	*Ethical concerns*	Some rewards could be harmful
		Privacy and bank statements
		Not freely entering treatment
		Some rewards could be harmful
		Withholding reward if client relies on it
	Incentives are or are not a bribe	
	It’s not about right and wrong	
***Conceptual and theoretical issues***	Could CM be disempowering?	
	Do gamblers need additional rewards?	
	Motivation	
	Rules	
	Schedule of reinforcement	
	Therapeutic relationship affected	
	Would CM reinforce gambling?	CM could reinforce similar patterns to gambling
		Don’t use prizes or words like prizes
		Gambling is different to alcohol & drugs
		Gambling linked to money and rewards
		Rewards with monetary value a concern
		Similarities between gambling and CM could help
		You don’t give a gambler money
***Barriers and challenges to implementation***	Clients concealing their problem	
	Clients feel unworthy of rewards	
	Clients could sell incentives to gamble	
	Clients might give reasons for missing sessions	
	Clients want evidence CM will work	
	CM causes conflict between clients	
	Coercion by family for rewards	
	Cultural barriers	
	I can’t see any barriers	
	Lockdown	
	Proving abstinence difficult	Bank statements helpful
		Client’s discomfort showing bank statements
		Deception bank statements
		Not everyone has access to bank statements
		Uncomfortable asking for bank statement
		Proving abstinence difficult-general comment
	Too much for clients to take in	
***Outcomes***	Addressing the underlying addiction	
	Attendance	
	CM could work for some clients	
	Cutting down goals an option	
	Help to establish abstinence	
	Long-term change	
	Uptake of blocking strategies	
***It’s about how you do it***	Admin do it or automate it	
	Addressing resistance unmotivated clients	
	Explaining CM well	
	Getting clients, staff and family on board	
	Getting the timing right	
	How you do it makes a difference	
	Managing multiple short episodes	
	What will happen in sessions matters	
***Political and society context***	Benefit to society	
	Media and society negative views	
	Political context for gambling in UK	

The Thematic Analysis used complete coding. All root codes are shown above as well as how they were initially categorised. Examples of child codes are shown for selected root codes

## Results

### Sample characteristics

Table 2 provides a summary of participant characteristics. Participants were counsellors, therapists, managers, trainers, supervisors, and frontline advisors working for a range of gambling services: National Health Service (NHS) specialist services, charity run providers of helpline, community, or residential treatment, Citizens Advice Bureau workers specialising in gambling problems, and practitioners in statutory addiction services or private practice.

**Table 2:** Sample characteristics

Characteristic	N	%
***Region***		
London	4	13.3
Midlands	6	20.0
SE England	8	26.7
SW England	1	3.3
Scotland	2	6.7
Wales	4	13.3
Yorkshire/Humber	5	16.7
***Service type***		
Addiction Services (alcohol, drugs, gambling)	2	6.7
Citizens Advice Bureau	4	13.3
GamCare	5	16.7
GamCare partner	11	36.7
NHS	4	13.3
Residential	2	6.7
Other	2	6.7
***Role***		
Frontline advisor	6	20.0
Practitioner, counsellor or therapist	16	53.3
Manager or senior clinician	8	26.7
***Age group***		
18–24	0	0.0
25–34	1	3.3
35–44	11	36.7
45–54	8	26.7
55–64	9	30.0
65–74	3	10.0
***Sex***		
Female	20	66.7
Male	10	33.3
***Ethnicity***		
Asian or Asian British	2	6.7
White British	21	70.0
Other White (including regions of British Isles)	6	20.0
Other non-white	1	3.3
Education (for example*)		
Level 2 (GCSE grade A*–C)	2	6.7
Level 3 (AS/A level)	2	6.7
Level 4 (cert of HE/ BTEC)	0	0.0
Level 5 (diploma)	7	23.3
Level 6 (bachelor’s degree)	10	33.3
Level 7 (master’s degree)	6	20.0
Level 8 (doctorate)	3	10.0
***Lived experience***		
Yes	5	16.7
No	25	83.3
***Years working in gambling field***		
2 or fewer	15	50.0
> 2–7 years	8	26.7
> 7–12 years	4	13.3
> 12 years	3	10.0
***Experience of incentives***		
No	23	76.7
Yes	7	23.3

The most common therapeutic interventions used were CBT (*n* = 18), MI (*n* = 11), and humanistic counselling (*n* = 10). Other approaches used by two to six practitioners were education about the brain and gambling, the recovery model, social behaviour network therapy, mindfulness-based 3^rd^ wave CBT approaches, psychodynamic and 12-step. Most practitioners drew on more than one model, often from a range of approaches. Few counsellors and psychotherapists described a strong allegiance to the model they had been trained in.

### Providers’ views of CM for gambling

Four themes were developed from the codes, with the pragmatic purpose of informing the development and implementation of a feasibility study of CM for gambling.

#### **Theme 1: Clash of philosophy, but “willing to be swayed”**

 Practitioners described experiencing a clash with their personal treatment philosophy centred around concepts of *personal motivation*, *natural rewards*, *self-responsibility*, and *empowerment* that were considered incompatible with providing incentives*.* Participants spoke about *motivation* as something internal to the person: a person needed to reach a point where *they want to change and are ready to change* (P16), where they *have personal motivation* (P20) where change would be *for themselves not for others* (P23). It was considered that recovery brought its own rewards both due to positive experiences in recovery and a sense of accomplishment (*n* = 9)*; they come back week on week saying, I've had another great week, now that is their reward (P2).*

Practitioners (*n* = 13) felt that CM could be disempowering. They expressed concern that the therapist takes a role where they are in control of purchases and rewards, activating a parent–child dynamic:

It also feels quite parental, like the counsellor and the service are providing the structure and it feels parental in terms of like, you know, pat on the head, well done kind of thing (P15).

Some (*n* = 5) also felt that clients need to be encouraged to take responsibility: *we encourage them to develop responsibility for what they're doing for themselves (P28).*

Others (*n* = 9) felt that CM was a good fit with the approach that they already take, both in promoting clients to apply principles of positive reinforcement by rewarding themselves and encouraging engagement in rewarding activities. Views from three participants put less emphasis on individual self-responsibility and more on the power of incentives to convey the message that people are there to support, encourage and recognise achievements of the client, potentially increasing a sense of self-worth and agency: *little tokens of recognition are so important for them because they feel they are useless (P11).*

Despite reservations, most of those who felt a clash with CM and their approach were open to exploring CM: *I’m willing to be swayed (P1).* Views became more positive with more information/reflection; the reverse was not observed. Participants (*n* = 12) spoke about the importance of conducting research into CM or the need for an evidence-base for using CM for gambling problems: *I don't think you should dismiss anything unless you tried it. (P10).* Overall, most participants supported trialing CM for gambling and would be willing to be involved in future trials, while six were unsure, and only two were decidedly against.

#### **Theme 2: CM could reinforce similar patterns of behaviour to gambling**

 Money was considered to be central to gambling (*n* = 9) in a way that it is not central in other addictions: *money reward is their drug* (P16). A person’s relationship to money could change through gambling to become unreal, *like monopoly money* (P17) or could be contradictory such as collecting tokens for a supermarket while losing large amounts of money gambling. The explanations of CM made it clear that money would not be given as an incentive, and practitioners agreed with this. Some participants shared the view that the use of prizes or the use of the term “prizes” is inappropriate for gamblers (*n* = 5), due to similarity with gambling.

More than half of the participants (*n* = 17) said that CM is like gambling (even if money and prizes are not used) because the person is doing something for a reward of monetary value: *if I do this, I will get that* (P10). The practitioners often felt that their work involved trying to break patterns of behaviour (e.g. using rewards to soothe difficult feelings), interrupt a certain mindset (e.g. something for nothing), and reduce the feelings associated with gambling (e.g. highs of wins). There was concern that CM could represent a similar system to gambling and reinforce these patterns of behaviour, thinking and feeling:

You've got someone that's moved away from getting some sort of cash for behaviours that are problematic and then we're almost reinforcing a system that is potentially similar to that… We actually want to just completely remove ourselves from that and change that pattern completely, so that there's no kind of monetary value attached to successful recovery. (P24).

Some (*n* = 3) also expressed concern that problematic dopamine pathways in the brain would be reinforced by CM: *could it create another dopamine rush?* (P19). It was felt that CM would conflict with approaches to treatment aimed at *re-establishing the cognitive function outside of the realms of reward* to redress *an over-exaggerated reward system (P16)*.

#### **Theme 3: The CM system could be manipulated, reducing trust between client and therapist**

 Concerns about implementing CM for gambling centred around the potential for clients to manipulate the system to gain money to gamble, or that some clients could be coerced by others to do so. Participants (*n* = 17) felt that deceptive behaviour could be expected from clients with gambling problems: *where there’s an urge there’s a way (P13*).

A key concern was that clients may sell the incentives to gamble (*n* = 15); vouchers were seen as *easily exchangeable* (P28), but other incentives might also be sold: *they would sell a sofa to get money to gamble* (P7). For one participant, it was a potential source of money to gamble that could be hidden from family members: *it would be a good way for them to get money without anyone knowing about it* (P19). Only one person described the view that if a minority chose to sell their incentive, then that is their choice: *they are adults with capacity* (P4).

Another concern identified by participants was the difficulty proving abstinence from gambling (*n* = 21). Some participants felt sharing bank statements would lead to greater transparency and accountability (*n* = 5). However, most identified numerous ways that a client could cheat the system if proof of abstinence relied on sharing bank statements: having multiple accounts, credit cards, prepaid credit systems, finding money, begging, borrowing, selling items for money to gamble, using someone else’s account, transferring an account to someone else’s name, PayPal, getting someone else to place bets for them, and forging bank statements: *a bank statement isn’t proof that someone’s not gambling by any stretch of the imagination (P27).*

A related concern (*n* = 7) was that incentives could lead to disagreements or friction between practitioner and client about whether the client was able to provide a valid explanation about expenditure or non-attendance. In the extract below, the counsellor described a way of working based on trust, while also being aware that the client could be adept at deception. By asking for evidence of abstinence, the stance of the counsellor would be changed. Several counsellors expressed a similar concern that the clients would be less likely to be open about their issues in this context (*n* = 8), such as being able to be open about underlying issues and relapses (*n* = 8).

[When] someone tells you their story and at any given time, well, that’s their story, and it may or may not be true, but you work with the story… if I start doing what other people are doing like asking to check their bank statements and things like that, I’m not sure then that they open up about some of the really difficult stuff that they’re carrying around. (P4)

#### **Theme 4: It’s about what you do and how you do it**

 Participants’ openness to CM varied depending on factors such as who receives the intervention, which behaviours are targeted, what the rewards are, and how it is implemented. Practitioners (*n* = 10) felt that CM could be helpful for specific groups of people, such as prisoners, disadvantaged and vulnerable clients, those with learning difficulties, and ethnic minorities. It was also felt that incentives might be more effective for a certain type of gambler: *it would probably depend on why they’re gambling (P29).* Some participants (*n* = 5) felt that clients should be selected for CM rather than taking a *one size fits all* (P27) approach, and that incentives need to be tailored to the individual based on an assessment or formulation of the client’s problems.

Eighteen participants held the view that providing incentives to clients could improve attendance of early appointments and groups. Common barriers to continuous attendance included *opening up about problems getting too painful* (P1), not feeling understood by the therapist, the treatment not being seen to be helping, fear of change and lack of faith in change, chaotic lifestyles, childcare or other dependents, and overconfidence. Although those engaged with incentives might be less motivated to change, these practitioners felt that it would be beneficial to work with resistance, educate, and increase awareness of available help: *If you get them through the door and you’ve got enough skills within your team, you can get them on that journey.(P7).*

Others felt that incentivizing attendance would not be helpful (*n* = 12), as people would be *just turning up* (P27), and this would be a waste of time and demotivating for practitioners. Fewer participants felt CM should target abstinence (*n* = 6), and some participants (*n* = 5) raised concerns that CM might exclude clients working towards cutting down gambling.

Participants often expressed views that rewards should be meaningful to the individual (*n* = 10), and most (*n* = 21) felt that rewards that promote personal interests and social activities would be welcome (e.g. health and fitness activities, movie tickets, restaurant vouchers, education, etc.). Six participants also commented on the level of the reward in the scenarios, suggesting this could be low and not salient to gamblers who *might have been gambling, 500 pounds, a thousand pounds a week (P12).* Participants also commented on the schedule of reinforcement; several expressed concern about reducing the value of rewards when goals are not reached (*n* = 8) as this might be experienced as failure. Others (*n* = 10) felt that there would be a risk the client might return to gambling when the incentives/rewards were withdrawn, and *would they stop coming?* (P5).

Practitioners also emphasised the importance of carefully planning the CM intervention and delivery: *I think it comes down to how it's implemented and delivered (P12).* It was seen as important to be able to get staff, clients, and family members on board (*n* = 11), so that they *believe in it and have faith in it* (P12). This might involve staff training, service user involvement in the design of the intervention, and involving family members in the intervention where possible. Key to this was explaining CM clearly, what the incentives would be, and what the client needed to do to get them (*n* = 3). The need to keep clear boundaries was also highlighted: *it [has to be] very clear because that is what they respond to, very clear boundaries (P9).*

A further concern (*n* = 10) was the amount of time that the implementation of CM would take: *it adds an extra layer of work* (P28). Some practitioners (*n* = 5) suggested that the CM aspect of treatment should be automated or that administration staff deal with the reward-exchange. The cost of CM was seen as another potential barrier to implementation (*n* = 16): i*n gambling, the agencies don't have a lot of money* (P24). Practitioners found it difficult to comment on the cost-effectiveness of CM given that the research base for CM for gambling is in its infancy, and their roles did not involve budget allocation. Some participants (*n* = 6) felt that the funding for CM could potentially come from the gambling industry.

## Discussion

The aim of this study was to consider the feasibility of CM as a treatment for GD and problem gambling by addressing the question: “What do providers of gambling treatment services perceive as potential benefits of, and barriers to, the implementation of CM for gambling?” Overall, four key themes characterizing treatment service providers’ perceptions of CM were developed from the data. The first theme was that there could be a clash with practitioners’ treatment philosophies, but that they would be willing to consider the use of CM if it was shown to be effective. Second, there were concerns that CM could reinforce similar patterns of behaviour to gambling. Third, there were concerns that the CM approach could be manipulated which may reduce trust between client and therapist. A final theme linked benefits of CM to how it is implemented in practice. Taken together, these themes broadly correspond with views identified in the context of CM for treatment of substance-use disorder [19–23] and extend them for the first time to a UK gambling treatment setting. The implications of the findings for the feasibility of CM for GD are discussed below.

Internationally, practitioners often view CM as conflicting with the philosophies they adhere to within their substance misuse treatment settings [21, 23, 24, 30, 35]. Theme 1 from the present study identified potential sources of conflict from UK-based gambling treatment service providers regarding CM and treatment philosophy. Specifically, participants raised concerns and issues related to personal motivation, natural rewards, self-responsibility, and empowerment. Taken together, these views could be seen as emphasising the importance of `intrinsic motivation’ over `extrinsic motivation’. Intrinsic motivation has been described as doing something naturally enjoyable or associated with experiences of autonomy and competence, while extrinsic motivation refers to doing something for a reward or to avoid punishment [36]. In the addiction treatment literature, both external and internal motivators are considered to play a role in promoting change [37]. Wong et al. [38] found that early abstinence in treatment for cocaine dependent outpatients (*n* = 126) predicted subsequent abstinence and self-efficacy as measured by the Situational Confidence Questionnaire. This suggests interventions that assist abstinence initiation early in treatment could potentially increase experiences of autonomy and competence (i.e. intrinsic motivation). However, the lack of agreed measures for intrinsic motivation and the questionable conceptual status of the construct may have hampered research into the impact of CM on intrinsic motivation in addiction treatment [39]. Further research is needed to understand the interplay of different types of internal and external reinforcers in the process of change in addiction, and how CM influences this process [32]. As the impact of CM on personal motivation was a key concern for gambling practitioners, it could be an important construct to measure in a future study of CM for gambling.

Despite conflicting philosophies, most practitioners valued research evidence and were open to changing their views if the evidence supported the use of CM for gambling. This supports the view that research evaluating the effectiveness of CM for gambling is called for. Furthermore, the active components of CM are not clear [24], and components such as goal setting with regular monitoring may contribute to the impact of CM [40]. Therefore, a study design that can isolate the impact of incentives from other aspects of the intervention would make a stronger case for CM if found to have benefit. Not all gambling practitioners perceived a clash with their approach; those who already use behavioural principles (e.g. achieving personal goals during recovery) were generally more aligned with the CM approach. This suggests there may be benefit in training practitioners in behavioural principles, or trialing CM where these are already used in practice [34].

Theme 2 highlights concerns that CM could reinforce patterns similar to an addiction have not been raised in substance misuse studies. Daar and Dixon [41] describe gambling as a “game of chance” with an unequal ratio of bets to payouts; from a behavioural perspective, a random ratio schedule of reinforcement, conditioned reinforcers, and verbal behaviour all contribute to gambling behaviour. The use of prize draws, which involve random or variable outcomes, in CM research for substance misuse [42] resembles gambling based on this definition. Interestingly, Petry et al. [42] found no difference in gambling behaviour during or after treatment when comparing stimulant users taking part in prize-based CM (*n* = 407) with a control group receiving usual care (*n* = 396). While there may be legitimate concerns about the use of prize draws with people with gambling problems, the extension to incentives/rewards that are clear and predictable seems unfounded, at least from a behavioural perspective. It is also worth noting that some practitioners have come to understand aspects of the neuroscience of gambling (related to dopamine and reward systems) [43], such that they conclude they should advise clients to avoid any reward-based actions during recovery. Neuroscience explanations of various psychological issues can be alluring even when they are not directly relevant [44]. Such views are reductionist and rather simplistic since they do not reflect the multi-layered nature of modern neuroscience involving genetic, epigenetic, endocrine, psychological, and behavioural levels. They may also inadvertently limit understanding of addiction by diverting attention from environmental-based treatments such as CM [45]. The frequency of this concern being raised suggests a need to further understand this perspective and educate practitioners about the nature of gambling from a behavioural perspective discriminating between intermittent rewards, and a healthy relationship with predictable rewards.

Theme 3 highlighted concern about clients selling incentives to fund addictions, which is also a common concern in substance misuse studies [21, 24]. However, broader concerns about deception to obtain incentives have been less emphasized elsewhere. Gamblers were commonly seen as likely to claim they are abstinent to gain incentives, falsifying bank statements, providing false excuses about why they cannot attend sessions, and be less open than they might be in sessions with therapists. The potential negative impact of deception, in the context of CM, on the therapeutic relationship is a key concern for some practitioners, and as the quality of the therapeutic relationship is linked to outcomes in addictions literature [46] this concern should be further explored. This raises questions about the suitability of incentive systems that have the potential to be manipulated. Future work might evaluate whether manipulation occurs, and the extent to which this has a negative impact on the implementation of CM.

We found that participants’ views of CM depended on how it might be implemented (Theme 4). There was greater support for targeting attendance early in treatment than abstinence from gambling, and more support for using a credit system towards recovery-related rewards than vouchers. In line with previous UK-based CM research, there was also significant support for tailoring the interventions to individuals or groups who might benefit the most [24]. Some practitioners commented that CM may be beneficial for clients seeking to reduce, but not stop, gambling on certain activities. Although further research is needed on the compatibility of CM with harm-minimisation practices such as ‘safe gambling’ [47], it is reasonable to assume that, like seen in substance use settings, CM is likely to be efficacious for those seeking to be abstinent from gambling rather than cutting down [42]. This issue notwithstanding, our findings show that considering the views of practitioners when designing interventions is likely to prompt less resistance from staff asked to implement the CM intervention.

Most of the evidence for CM for substance misuse suggests that CM has a strong impact during treatment, but that treatment effects may diminish after the incentives are withdrawn [11, 48]. Practitioner concerns about short-term impact of CM points to the need for a greater understanding of long-term recovery and how this can be sustained. CM might be most effective if coupled with interventions designed to promote ongoing contact with positive reinforcement in recovery. For example, a programme for cocaine-dependent outpatients (*n* = 64) that combined the Community Reinforcement Approach with CM found treatment effects were maintained six months after completion of the voucher programme [49].

Most practitioners recognised that funding for CM (incentives and practitioner time) would not be readily available in their organisation, and some suggested additional funds for CM could come from the gambling industry, which already funds much of UK treatment for gambling problems. Perceived cost needs to be addressed at a higher organisational and treatment system level and being able to demonstrate cost-effectiveness will aid the case for more widespread adoption of CM for gambling.

### Limitations

This study recruited 30 practitioners from a wide range of gambling treatment services in the UK. As a result, findings may differ by country, context, and whether practitioners were drawn from gambling treatment services or other types of service who see gamblers. Although the sample size was adequate to identify a range of common views, numbers expressing different views should not be seen as quantitatively representative of the wider population of gambling treatment practitioners. To address this, a survey measure, based on the interviews and expert consultation, is under development by our group to assess these views within UK treatment service settings. The perspectives of other relevant stake-holders such as people with lived experience of gambling problems were not included in this paper and also merit investigation.

## Conclusions

There is support from UK practitioners for research on CM for gambling, despite most experiencing conflicts between CM and their current beliefs about practice. Increased education about behavioural principles (e.g. positive reinforcement) and application to the treatment of addictive behaviour may help reduce perceived conflicts. To address practitioner concerns, future research should seek to understand how CM for gambling influences processes of change (e.g. engagement in treatment, motivational change, and long-term recovery) as well as outcomes such as attendance, abstinence, and progress in recovery. Practitioner views of how CM are implemented (i.e. the `who, what, and how’) can also inform implementation of CM to minimise resistance and potential abuse of the CM approach.

## Supplementary Information

**Additional file 1:** Interview Topic Guide.

## Data Availability

The datasets are not available due to the sensitive nature of the study, as they contain confidential information that could compromise participant confidentiality and consent.

## Abbreviations

CBT: Cognitive behavioural therapy
CM: Contingency management
CMAA: Contingency Management Adoption Attitudes
ATT-CM: Contingency management for attendance
ABS-CM: Contingency management for abstinence
GD: Gambling disorder
MI: Motivational interviewing
NHS: National Health Service
PSI: Provider survey of incentives
TA: Thematic analysis
UC: Usual care
