# Supplementary material for: Gambling treatment service providers’ views about contingency management: a thematic analysis
Source: Harm Reduct J. 2022 Feb 25;19:19. doi: 10.1186/s12954-022-00600-0 (PMC8876078; doi:10.1186/s12954-022-00600-0)
Supplement: Supplementary file 1 — Additional file 1: Interview Topic Guide. [file 12954_2022_600_MOESM1_ESM.docx]

## **Supplementary Material**

***Gambling treatment service providers’ views about contingency management: A thematic analysis***

## **Topic Guide**

# Introduction (5 minutes)

**Aim: to introduce the research and set the context for the interview**

1. Introduce self and role at Swansea University
2. Introduce the study and what it is about (check they have also read and understand the PIS)
3. Purpose and length of the interview
4. Voluntary participation, right to stop to have a break and to withdraw
5. Explain confidentiality and limits
6. Discuss how participants could seek support if interviews have an emotional impact
7. Audio-recording session and right to request recording stops or pauses
8. Any questions or concerns
9. Record consent

# Background information (10 minutes)

**Aim: to collect data that provide context for the interview**

Structured Questions will be visible in PowerPoint on the shared whiteboard within Zoom or Teams, answers can be given verbally and recorded by the researcher or completed in Qualtrics survey software.

1. Age
2. Sex
3. Gender
4. Ethnicity
5. Professional role
6. Qualifications
7. Lived experience of gambling
8. Years spent working in the field
9. Experience of using incentives in practice.

Participants will be asked to expand on their work role, describe the types of interventions they use with clients, and their own preferred models of working.

# Description of contingency management (5 minutes)

**Aim: to ensure understanding of Contingency Management (CM), and how it might be applied to gambling**

1. Provide the below explanation of CM.

Contingency management (CM) is an intervention for promoting behaviour change that can be added to existing therapies for issues such as substance use disorders, medication adherence, smoking cessation, breastfeeding, and other health areas. CM interventions in, for example, substance use disorders most often involves the provision of tangible reinforcement, such as vouchers, in return for submission of drug negative urine samples. The delivery of a reward following the target behaviour results in an increased rate of that behaviour; for example the target behaviour of attending a treatment intervention would be expected to increase, or the target of abstinence could be maintained. Incentive programs usually involve giving the client his or her choice of prizes, services, or privileges (e.g. vouchers for goods such as food or credits towards the purchase of chosen items) when and only when the person meets a therapeutic goal. Clients know in advance that they can earn these incentives and what they must do get them. Therapeutic goals are broken down into steps so that any one step toward the goal is not too difficult for the client to achieve. Each step also must be defined so that it can be objectively verified to minimize disagreement and misunderstandings. Multiple trials of contingency management for drug misuse have been conducted, which constitute the largest single body of evidence for the effectiveness of psychosocial interventions in drug misuse. We do not know whether the same approach is effective for gambling, which is why we are carrying out research in this area

**Semi-structured interview (15 minutes)**

**Aim: to gain the participants perspective on the CM approach applied to gambling.**

1. In your opinion, do you feel this could be a successful approach in treatment for people who want help for their gambling problem?
2. Would you be interested in delivering such a programme if it became available?
3. What type of barriers to success do you think this type of approach might encounter?
4. Scenario 1: Throughout a 3-month program every time a client shares a bank statement clear of gambling activity (weekly), he or she receives credits that are exchangeable for goods or services. Each credit is worth one pound. The first clear statement earns 3 credits. If the client continues to stay abstinent, each subsequent clear bank statement increases the number of vouchers earned by 1, i.e., second clear in a row= 4 credits, third = 5 credits. A bank statement indicating unexplained expenditure or failure to submit a statement resets the value of the credits to the initial 3-credit position. No cash is given. Staff members assist in the exchange of credits for items. Clients may choose from a wide variety items. Some examples may include health club memberships, movie passes, gift certificates to local restaurants, etc. Therapists approve only those exchanges that support gambling-free prosocial activities, then someone from the clinic orders or purchases the item. What do you see as the strengths and weaknesses of this approach? (based on Kirby 2006)
5. Scenario 2: A client presents to a gambling treatment service for the fourth time, asking for help. Each time he or she presented in the past they did not engage for more than two sessions. The client is offered a six week, weekly intervention which will include a reward for attendance. Each session attended is rewarded with a voucher that can be exchanged for food or other goods. The first session attended is rewarded with a five pound voucher, the second consecutive attendance increases to six pound voucher, and so on. If a session is missed then the reward begins again at a value of five pounds. What do you see as the strengths and weaknesses of this approach?

# CM attitudes and beliefs -structured surveys (10 minutes)

**Aim: to field test these surveys with providers of gambling services, and identify the prevalence of specific beliefs in the study group**

Questionnaires will be visible on the shared whiteboard within Zoom or Teams, answers will be given verbally and recorded by the researcher, or completed in Qualtrics survey software.

1. Provider Survey of Incentives
2. Contingency Management Adoption Attitudes

# Semi-structured interview continued (10 minutes)

**Aim: to gain the participants perspective on the CM approach applied to gambling.**

1. Probe answers to PSI and CMAA which were the most extreme: you rated Q.—as ------, can you say more about what made you decide to rate it this way?
2. Probe answers to financial questions and how they relate to the service they work in.
3. Probe answers to philosophy questions and how they relate to their personal philosophy regarding gambling problems and treatment.

# Ending the interview (5 minutes)

1. Address any unmet needs identified in the interview by signposting to appropriate resources
2. Upon interview closure all interviewees will be asked if they believe there are any topics we may have missed that are relevant to this project or that they feel are of importance for discussion / consideration – any such topics brought forward will be included in our topic guide to be further explored with subsequent participants; as such the topic guide is an organic tool that will be used to facilitate interview discussions
3. Thank the participant for taking part.

**Structured Surveys**

**Provider Survey of Incentives for Gambling Treatment (PSI-GAM)**

Please use your experience and opinions to honestly respond to the following questions.

|  | Strongly disagree | disagree | Neutral | Agree | Strongly  agree |
| --- | --- | --- | --- | --- | --- |
| 1. Overall, I would be in favour of adding an incentive program to my treatment program |  |  |  |  |  |
| 1. If you give a tangible incentive to clients who’ve earned them, but not to others, it will result in clients arguing about rewards |  |  |  |  |  |
| 1. Most clients would sell the tangible incentives they receive. |  |  |  |  |  |
| 1. Clients who sell their tangible incentives will use the money to continue their gambling. |  |  |  |  |  |
| 1. Tangible incentive programs that cost £150 per client per month are worth it considering how effective they are. |  |  |  |  |  |
| 1. My treatment facility could not find funds for tangible incentives that cost £150 per client per month. |  |  |  |  |  |
| 1. Tangible incentive programs that cost £50 per client per month are worth it considering how effective they are. |  |  |  |  |  |
| 1. My treatment facility could not find funds for tangible incentives that cost £50 per client per month. |  |  |  |  |  |
| 1. Tangible incentive programs that cost £10 per client per month are worth it considering how effective they are. |  |  |  |  |  |
| 1. My treatment facility could not find funds for tangible incentives that cost £10 per client per month. |  |  |  |  |  |
| 1. Tangible incentives are worthwhile because they can get clients in the door for treatment. |  |  |  |  |  |
| 1. If a client is abstinent just to get the incentive, it could hurt the treatment process |  |  |  |  |  |
| 1. Many clients will see rewards for abstinence as cheesy or artificial. |  |  |  |  |  |
| 1. Incentives are just not right because they are rewarding the client for what he/she should be doing in the first place. |  |  |  |  |  |
| 1. Overall, incentives are good for the client/counsellor relationship |  |  |  |  |  |
| 1. Overall, incentives have negative effects on the client/counsellor relationship. |  |  |  |  |  |
| 1. Incentives are more likely to have positive effects on the client than they are to have negative effects. |  |  |  |  |  |
| 1. Incentives are more likely to have negative than they are to have positive effects on clients |  |  |  |  |  |
| 1. Incentives will cause jealousy among clients who do not get them. |  |  |  |  |  |
| 1. It would not be right to give an incentive for goals such as attendance if they are not proving they are abstinent from gambling. |  |  |  |  |  |
| 1. It would not be right to give an incentive to clients for not gambling when they are not fulfilling other treatment goals, such as attending a group. |  |  |  |  |  |
| 1. Incentives are useful if they reward clients for fulfilling treatment goals other than just providing evidence of not gambling, such as for regular attendance. |  |  |  |  |  |
| 1. The best incentive programs reward clients only when they are fulfilling multiple treatment goals (e.g. attending and abstinent to get a reward) |  |  |  |  |  |
| 1. The best incentive programs reward clients for only one treatment goal at a time (e.g., attending a group meeting) |  |  |  |  |  |
| 1. Incentive programs that require appointments with clients twice a week are not practical because most programs do not offer appointments twice a week. |  |  |  |  |  |
| 1. Incentive programs that require proof of abstinence at least once a week are not practical because most programmes do not routinely do this. |  |  |  |  |  |
| 1. Incentives help the client achieve abstinence from gambling, allowing the counsellor to focus on helping them make other life changes. |  |  |  |  |  |
| 1. Incentive programs are not consistent with my philosophy of treatment |  |  |  |  |  |
| 1. Incentives will stop the client from seeing beyond the external reward and prevent them from realizing their internal motivation. |  |  |  |  |  |
| 1. Incentives are a bribe. |  |  |  |  |  |
| 1. Abstinence will only last for as long as the incentives are given |  |  |  |  |  |
| 1. Giving incentives for verified abstinence from gambling helps the client to become abstinent |  |  |  |  |  |
| 1. Giving incentives for treatment attendance will not improve attendance. |  |  |  |  |  |
| 1. An advantage of incentive programs is that they focus on what is good in the client’s behavior (i.e., the ability to become abstinent), not what went wrong in their recovery. |  |  |  |  |  |
| 1. Consistently providing the client with incentives is likely to push the client back into denial. |  |  |  |  |  |
| 1. Any source of abstinence motivation, not just internal motivation, is a good thing for treatment. |  |  |  |  |  |
| 1. Incentive programs that require close tracking of client behaviour are too labour intensive to incorporate into our programme. |  |  |  |  |  |
| 1. Incentives are not useful for short-term treatments (e.g., 1 month or less) |  |  |  |  |  |
| 1. There are enough rewards in being abstinent; incentives are not necessary. |  |  |  |  |  |
| 1. Incentives do not address the underlying issues of addiction. |  |  |  |  |  |
| 1. Incentives can be useful whether or not they address the underlying issues of addiction |  |  |  |  |  |
| 1. I wish I could provide our clients with incentive rewards, but I don’t see how it’s affordable. |  |  |  |  |  |

***Contingency Management Adoption Attitudes* (CMAA)**

 Please use your experience and opinions to honestly respond to the following questions.

|  | Strongly disagree | disagree | Neutral | Agree | Strongly  agree |
| --- | --- | --- | --- | --- | --- |
| It is okay for patients to have the opportunity to earn prizes worth as much as £100 for abstinence |  |  |  |  |  |
| It is okay to pay patients for attending treatment |  |  |  |  |  |
| Incentives can have a positive effect on the patient/counsellor relationship |  |  |  |  |  |
